# Quantitative Measurement of the Thyroid Uptake Function of Mouse by Cerenkov Luminescence Imaging

**DOI:** 10.1038/s41598-017-05516-5

**Published:** 2017-07-18

**Authors:** Chien-Chih Ke, Zi-Ming He, Ya-Ju Hsieh, Chia-Wen Huang, Jia-Je Li, Luen Hwu, Yi-An Chen, Bang-Hung Yang, Chi-Wei Chang, Wen-Sheng Huang, Ren-Shyan Liu

**Affiliations:** 1 Biomedical Imaging Research Center, National Yang-Ming University, Taipei, Taiwan; 2 Department of Biomedical Imaging and Radiological Sciences, National Yang-Ming University, Taipei, Taiwan; 3 Molecular and Genetic Imaging Core/Taiwan Mouse Clinic, Animal Consortium, Taipei, Taiwan; 4 Department of Medical Imaging and Radiological Science, Kaohsiung Medical University, Kaohsiung, Taiwan; 5 Department of Nuclear Medicine and National PET/Cyclotron Center, Taipei Veteran General Hospital, Taipei, Taiwan; 6 Institute of Clinical Medicine, National Yang-Ming University, Taipei, Taiwan; 7 Department of Nuclear Medicine, School of Medicine, National Yang-Ming University, Taipei, Taiwan; 8 Biophotonic and Molecular Imaging Research Center, National Yang-Ming University, Taipei, Taiwan; 9 Division of Nuclear Medicine, Department of Medical Imaging, Cheng Hsin General Hospital, Taipei, Taiwan

## Abstract

Cerenkov luminescence imaging (CLI) has been an evolutional and alternative approach of nuclear imaging in basic research. This study aimed to measure the ^131^I thyroid uptake of mouse using CLI for assessment of thyroid function. Quantification of ^131^I thyroid uptake of mice in euthyroid, hypothyroid and hyperthyroid status was performed by CLI and γ-scintigraphy at 24 hours after injection of ^131^I. The ^131^I thyroid uptake was calculated using the equation: (thyroid counts − background counts)/(counts of injected dose of ^131^I) × 100%. Serum T4 concentration was determined to evaluate the thyroid function. The radioactivity of ^131^I was linearly correlated with the CL signals in both *in vitro* and *in vivo* measurements. CLI showed a significant decrease and increase of ^131^I thyroid uptake in the mice in hypo- and hyperfunctioning status, respectively, and highly correlated with that measured by γ-scintigraphy. However, the percent thyroid uptake measured by CLI were one-fifth of those measured by γ-scintigraphy due to insufficient tissue penetration of CL. These results indicate that CLI, in addition to nuclear imaging, is able to image and evaluate the ^131^I thyroid uptake function in mice in preclinical and research settings.

## Introduction

Radioiodine has long been used for thyroid studies^1, 2^. Radioactive iodine uptake (RAIU) and thyroid scan are commonly used to evaluate patients with suspected thyroid disorders. RAIU that measures iodine metabolism in the thyroid gland is based on physiologic incorporation of radioiodide into the thyroid gland and is followed by determination of the fraction of the dose taken up in the gland over a given time period^2^. It has been widely used to assess the thyroid function in clinical practice. RAIU along with a thyroid scan is useful in differentiating the causes of hyperthyroidism, such as Graves’ disease, Plummer’s disease and subacute thyroiditis^2^. However, these nuclear medicine procedures are seldom applied on small animals in the preclinical studies due to the drawbacks of low spatial and temporal resolution of nuclear imaging and relatively high cost of instruments^1, 2^.

Cerenkov luminescence (CL) has been used recently in biomedical applications of imaging with clinically relevant medical isotopes^3, 4^. CL is a phenomenon which was first described by Pavel Alekseyevich Cherenkov in 1934^5^. Charged particles traveling faster than the speed of light in a medium transfer their kinetic energy through interactions with the surrounding dipoles, in biological tissues mostly with water^5^. The randomly oriented water molecules will align with the passing super-relativistic charged particle and relax by releasing the transferred energy in the form of light^5, 6^. Cerenkov luminescence imaging (CLI) can be done using a sensitive camera optimized for low light condition which has a better resolution than any other nuclear imaging modality^3^. CLI has emerged quickly in preclinical molecular imaging with the use of various medically relevant radioisotopes, including ^15^O, ^13^N, ^68^Ga, ^89^Zr, ^64^Cu, ^225^Ac, ^90^Y, ^131^I, ^124^I, ^18^F and ^74^As, showing that the emitted radioactivity correlated well with the light output^7–10^.

With the development of optical imaging techniques and the improvement of its equipped CCD systems, the sensitivity and accuracy for CL detection has made this method highly applicable and competent in preclinical basic research setting^11^. In addition, advantages such as lower cost than that of nuclear imaging modalities, without setup of radiochemistry facilities, nearly identical imaging procedures and short image acquisition time of radionuclide-based optical CLI have provided an alternative and potential approach for researchers who are not majored in the nuclear imaging but need to conduct the radionuclide-based research.

The preclinical use of CLI in the study of radioiodine uptake are becoming more common in recent years. Jeong *et al*. successfully showed the CLI and nuclear imaging of ^131^I and ^124^I uptake by mouse thyroid gland and by NIS-expressing thyroid cancer cells^12^. Using ^99m^Tc-pertechnetate, Boschi *et al*. demonstrated CLI of the thyroid glands and salivary glands of mice^13^. Spinelli *et al*. reported the first ^131^I CLI of a human thyroid gland with a therapeutic dose of ^131^I for the treatment of hyperthyroidism^14^. Hu *et al*. demonstrated the application of Cerenkov luminescence tomography for evaluating the ^131^I uptake function of mouse thyroid^15^. These results have shown the translational potential of CLI.

To the best of our knowledge, there is no report showing that CLI is able to quantitatively measure the thyroid uptake of radioiodide. In this study, we have approved that CLI is feasible to measure the ^131^I thyroid uptake in mice in different status of thyroid function.

## Materials and Methods

### Mouse model of hypothyroidism and hyperthyroidism

The animal manipulation and experiment procedures were reviewed and approved by the Institutional Animal Care and Use Committee of National Yang-Ming University. Six week-old Balb/c male mice, purchased from National Laboratory Animal Center (NLAC, Taiwan), were housed in a temperature-controlled room with a 12-hour light-dark cycle and given access to food and water *ad libitum*. For induction of hypothyroidism, the mice were orally administered with methimazole (MMI, Sigma-Aldrich), 64 mg/kg in 200 μl distilled water, via gavage for 15 consecutive days. For induction of hyperthyroidism, the mice were intramuscularly injected with 2 μg of recombinant human thyroid stimulating hormone (rhTSH, Thyrogen, thyrotropin alfa, Genzyme) in 100 μl distilled water twice on two consecutive days. The control mice were administered with the same volumes of distilled water. Measurement of serum total T4 was carried out by using a Mouse/Rat Thyroxine ELISA kit (GenWay Biotech. Inc., San Diego, CA, USA) to assess the thyroid function.

### Correlation of CL signals and ^131^I activity *in vitro* and *in vivo*

To assess the correlation of CLI signals and radioiodide activity *in vitro*, ^131^I was serially diluted into 500, 250, 125, 63, 32 and 16 μCi (18.5, 9.3, 4.6, 2.3, 1.2 and 0.6 MBq) in 200 μl saline in Eppendorf tubes. CLI was carried out using an IVIS 50 imaging system (Perkin Elmer, Waltham, MA, USA) with a luminescence imaging setting of binning: 8, FOV: 12, f stop: 1, exposure time: 300 s. The signal of ^131^I-emitted CL was analyzed using Living Imaging Software (Perkin Elmer) and shown in p/s/cm^2^/sr.

To evaluate the correlation of injected ^131^I dose and CL signals in mouse thyroid, serial doses of ^131^I (0, 0.6, 1.2, 2.3, 4.6, 9.3 and 18.5 MBq in 200 μl saline) were intraperitoneally injected into mice followed by CLI 24 hours later. Before imaging, the mice were anesthetized by inhalation of 2% isofluorane mixed with oxygen. Quantification of CL signals from mouse thyroid was performed following the settings as described in *in vitro* study.

### ^131^I thyroid imaging and measurement of thyroid uptake function by γ-scintigraphy and CLI

^131^I thyroid imaging and measurement of thyroid uptake function were carried out on six euthyroid mice, six mice in hypothyroid status and six in hyperthyroid status. The mice were intraperitoneally injected with 18.5 MBq of ^131^I, followed by γ-scintigraphy and CLI 24 hours later. γ-scintigraphy was performed by placing animals prone at a distance of 6 cm under a 4-mm pinhole collimator equipped on a gamma camera (Symbia E, Siemens). Image was acquired for 30 min. CLI was performed subsequently using an IVIS 50 imaging system with the same luminescence imaging settings mentioned above. The radioactivity of the thyroid gland was obtained from the region-of-interest (ROI) drawn along 85% isocount contour of the thyroid gland over the anterior neck. The background activity was measured over the head with a ROI of the same size as the thyroid gland. The photon flux of the CL of the thyroid gland and the background was obtained using the similar method as γ scintigraphy. The percent thyroid uptake of ^131^I was calculated using the equation ((thyroid counts − background counts)/counts of injected ^131^I dose × 100%). Correction of the decay of radiotracer was done for each measurement.

### Statistical analysis

The numerical data were reported as means ± standard deviation (S.D.). Student’s t test was applied for comparison of serum T4 concentration and percent ^131^I thyroid uptake of each group with different thyroid function status. A significant difference was considered if the p value was less than 0.05.

### Ethical approval

All applicable international, national, and/or institutional guidelines for the care and use of animals were followed. This article does not describe any studies with human participants performed by any of the authors.

## Results

### Correlation of CLI signals and ^131^I activity *in vitro* and *in vivo*

**Figure 1:**
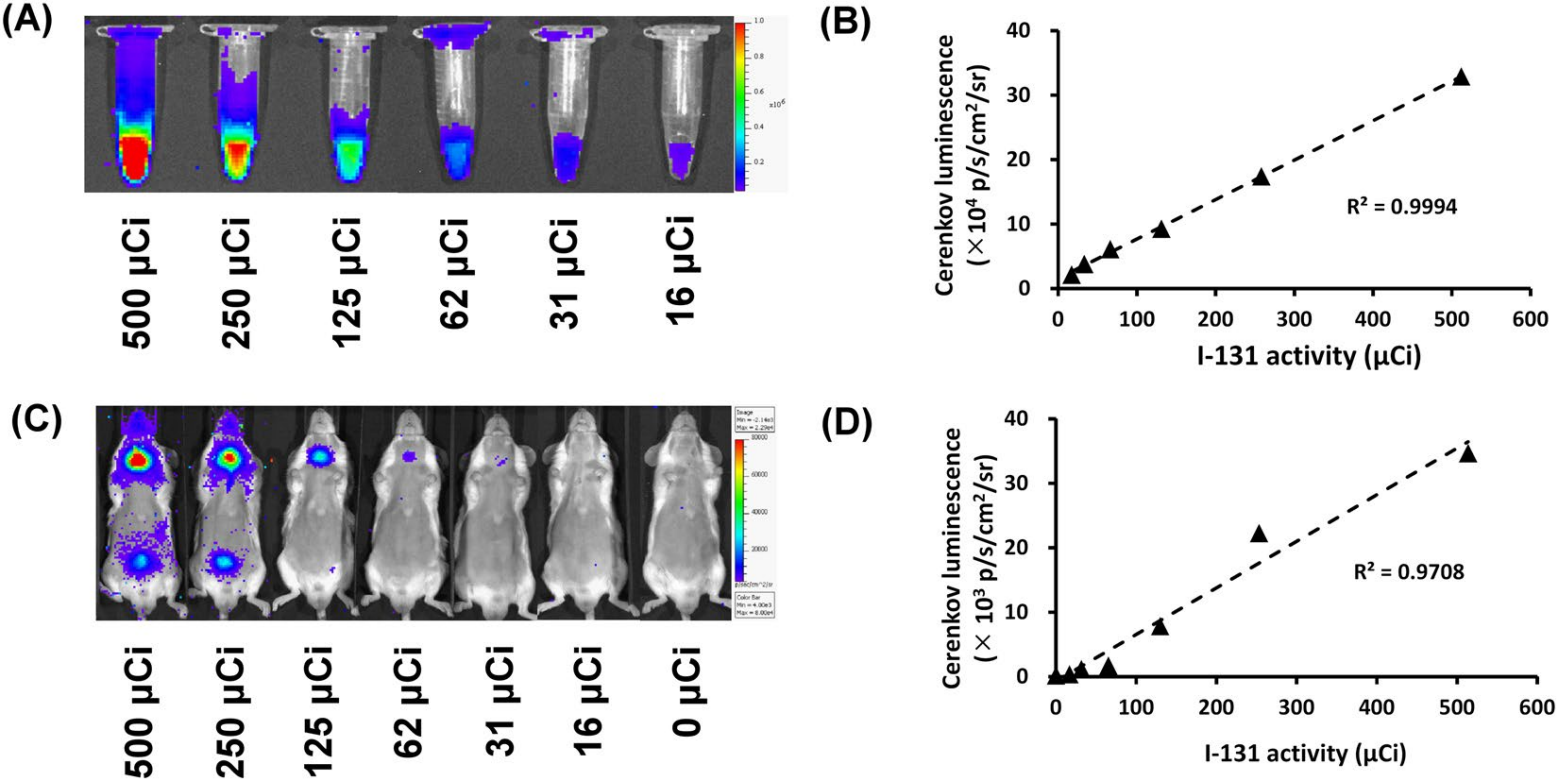
*In vitro* and *in vivo* CLI of ^131^I. CLI of ^131^I with the doses ranging from 16 μCi to 500 μCi (0.6 to 18.5 MBq) in Eppendorf tubes was performed using an IVIS 50 luminescence imaging system (**A**). The CL signals of ^131^I in each tube (shown as p/s/cm^2^/sr) were linearly correlated with the radioactivity (**B**). CLI of the mice at 24 hours after receiving ^131^I with the doses ranging from 0 μCi to 500 μCi (0 to 18.5 MBq) (**C**). CL signals from the thyroid and the radioactivities of the administered ^131^I revealed a high linear correlation (**D**).

^131^I with activity ranging from 500 to 16 μCi (18.5 to 0.6 MBq) was aliquoted in the same volume (200 μl saline), placed in Eppendorf tubes, and then imaged by CLI using the IVIS 50 system. The luminescence intensity of ^131^I corresponded well with the doses of radiotracer (Fig. 1A). Within a ~5 cm^2^ ROI, 500 μCi (18.5 MBq) and 16 μCi (0.6 MBq) of ^131^I resulted in signal of approximately 3 × 10^5^ and 2 × 10^4^ p/s/cm^2^/sr, respectively. A high correlation (R^2^ = 0.9994) was observed between the luminescence intensities and ^131^I activities (Fig. 1B).

To determine whether the accumulation of different doses of ^131^I in the thyroid gland of healthy mice can be accurately measured by CLI, doses of ^131^I from 0 to 500 μCi (0 to 18.5 to MBq) were intraperitoneally injected into the mice and CLI was performed 24 hours later. The results showed that the CL emitted from the thyroid gland was visually correlated with the amount of ^131^I activity injected (Fig. 1C). The signals from the thyroid ROI were ranging from 3 × 10^4^ p/s/cm^2^/sr (18.5 MBq ^131^I) to 4 × 10^2^ p/s/cm^2^/sr (0.6 MBq ^131^I). The CL signals were well correlated with the ^131^I doses (R^2^ = 0.9708) (Fig. 1D). The results suggested that the minimum dosage of ^131^I CLI imaging of mouse thyroid gland is 125 μCi (4.6 MBq).

### *In vivo*^131^I CLI and γ-scintigraphy of the mice in different thyroid function status

**Figure 2:**
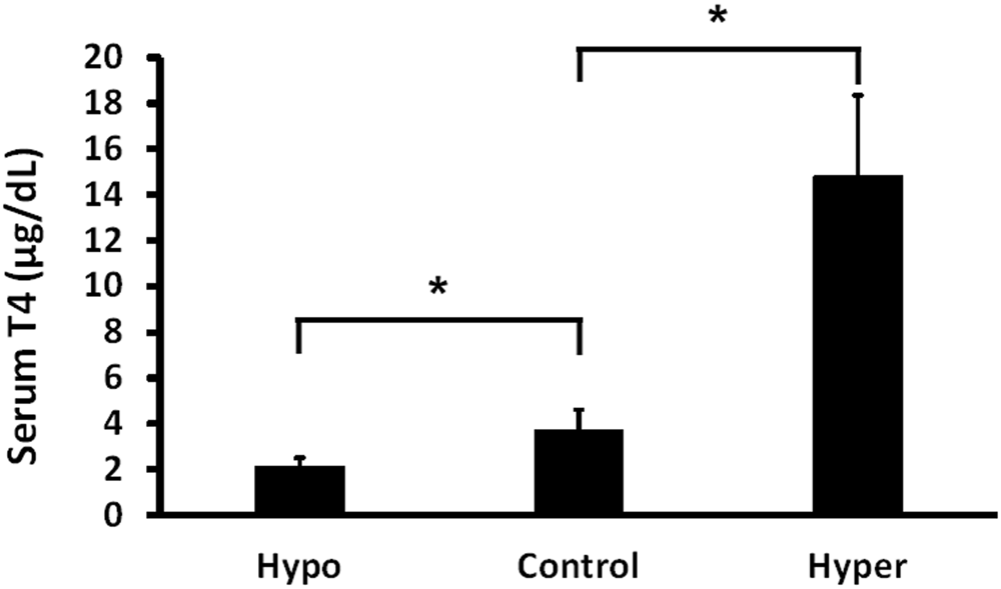
Serum T4 level in mice after induction of hypothyroidism (Hypo) by treatment of methimazole and induction of hyperthyroidism (Hyper) by rhTSH (Thyrogen). The results showed a significant decrease and increase of serum T4 in mice in hypo- and hyperfunctioning thyroid status, respectively, as compared with the control animals. (Student’s t-test, *p < 0.05).

Mouse models of hypothyroidism and hyperthyroidism were established by treatment with MMI and Thyrogen, respectively. Total serum T4 was measured to confirm the successful induction of hypothyroidism and hyperthyroidism. The results showed that the serum T4 level was significantly higher in rhTSH-treated mice (~14.8 ± 3.5 μg/dl) and significantly decreased in MMI-treated mice (~2.1 ± 0.4 μg/dl), as compared to that in the euthyroid subjects (~3.7 ± 0.9 μg/dl) (*p* < 0.05) (Fig. 2).

**Figure 3:**
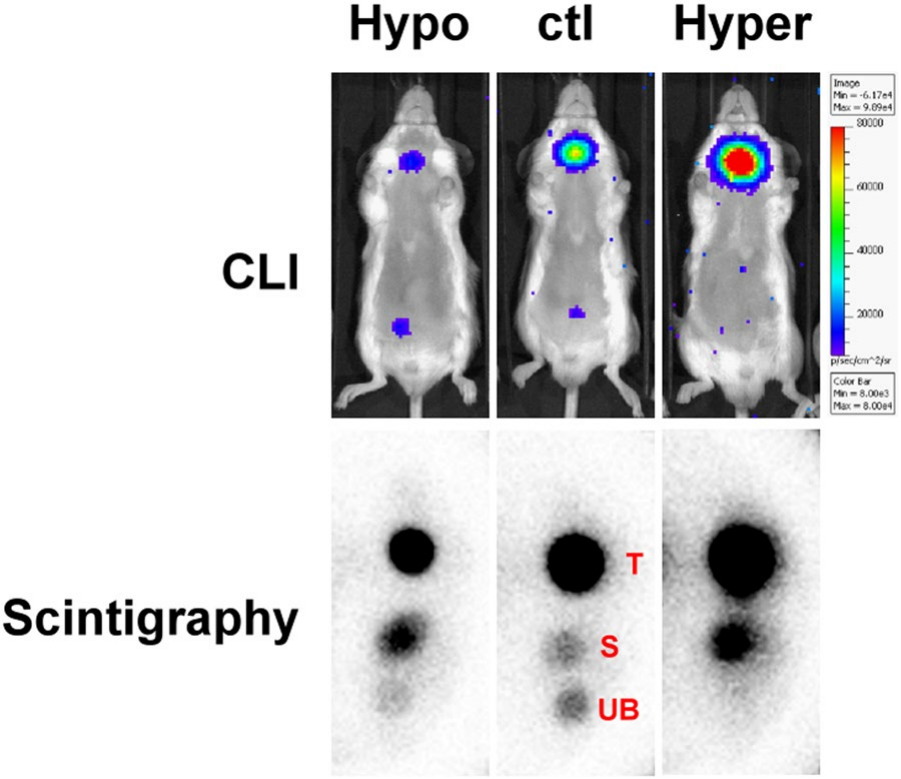
^131^I γ-scintigraphy and CLI of the mice in hypothyroid, euthyroid and hyperthyroid status. The CL signals emitted from the stomach(s) were unable to be demonstrated by CLI due to the attenuation effect of the soft tissue overlying the stomach and of the food in the stomach. (hypo: hypothyroid; ctl: control euthyroid; hyper: hyperthyroid; T: thyroid gland; S:stomach; UB: urinary bladder).

**Figure 4:**
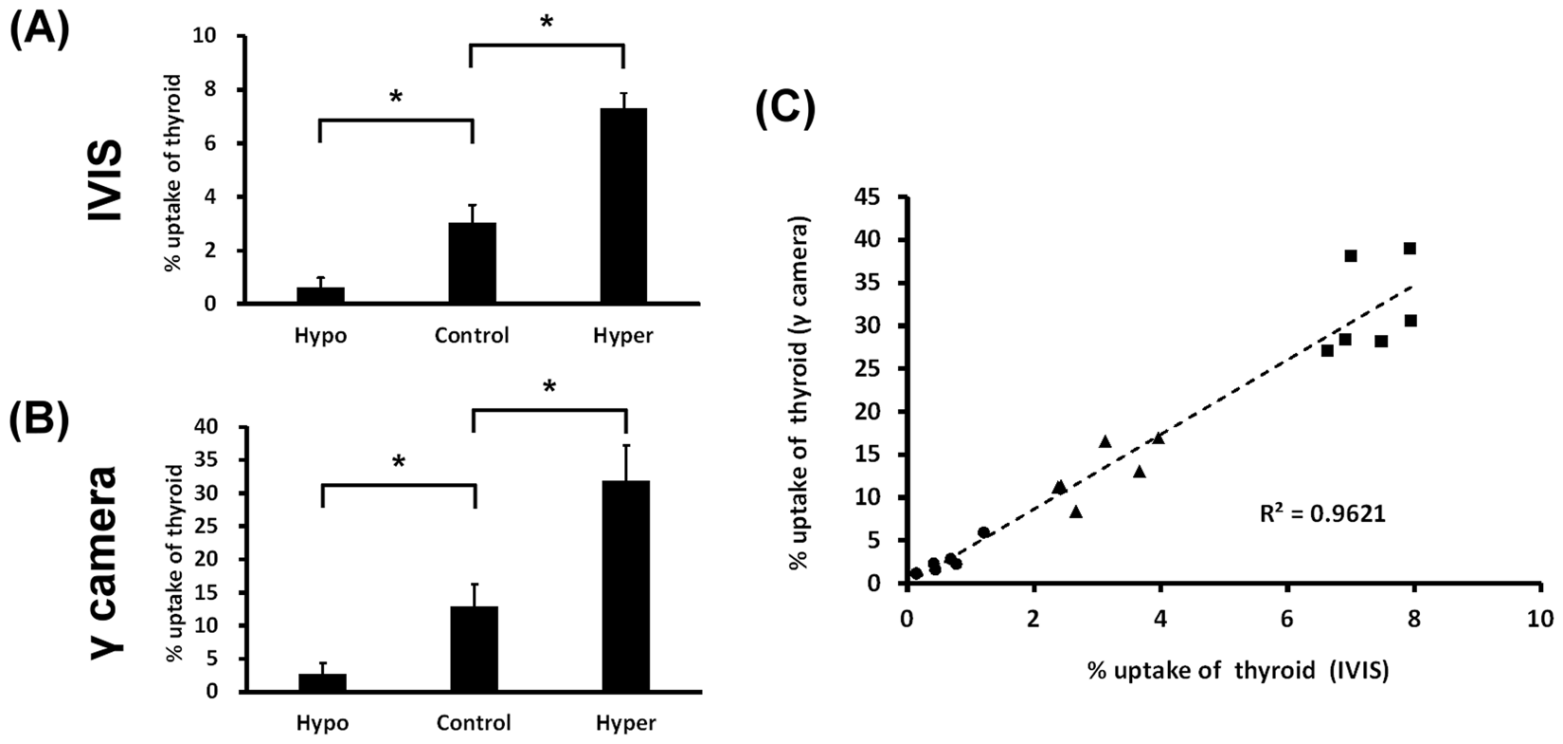
Percent ^131^I thyroid uptake measured by CLI (**A**) and γ-scintigraphy (**B**). A significant decrease and increase in percent ^131^I thyroid uptake were observed in mice with hypothyroidism (Hypo) or hyperthyroidism (Hyper), respectively, as compared with the euthyroid mice (control). (**C**) A high linear correlation was observed between the percent uptake values as measured by CLI and γ-scintigraphy. The percent uptake values of the hypothyroid mice (◾), of the hyperthyroid mice (•) and of the euthyroid mice (▲) had no overlap in the XY plot. (Student’s t-test, *p < 0.05).

The mice in hypothyroid or hyperthyroid status were intraperitoneally administrated with 500 μCi (18.5 MBq) of ^131^I, followed by CLI and γ-scintigraphy 24 hours later. The CL images showed obvious reduction or increase of luminescence signals in the thyroid glands in hypothyroid or hyperthyroid status, respectively, as compared to the euthyroid mice (Fig. 3). ROI analysis showed that the CL signals were 2 × 10^3^ ~ 2 × 10^4 ^p/s/cm^2^/sr for the hypothyroid mice, 1.2 ~ 1.4 × 10^6 ^p/s/cm^2^/sr for the hyperthyroid mice and 4 ~ 7 × 10^5 ^p/s/cm^2^/sr for the control euthyroid mice. A similar result was observed by γ-scintigraphy, showing reduced or increased γ signal in the thyroid glands of mice in hypothyroid or hyperthyroid status, respectively (Fig. 3). The radioactivity (counts per second, cps) obtained from the ROIs of the thyroid glands were 10 ~ 50 cps for hypothyroidism, 200 ~ 300 cps for hyperthyroidism and 70 ~ 130 cps for euthyroid mice. The percent ^131^I uptake of the thyroid measured by CLI were 0.6 ± 0.3%, 3.0 ± 0.6% and 7.3 ± 0.6% for hypothyroid, euthyroid and hyperthyroid status, respectively. By γ-scintigraphy, the percent ^131^I uptakes of thyroid were 2.7 ± 1.7%, 12.9 ± 3.3% and 31.9 ± 5.3% for hypothyroid, euthyroid and hyperthyroid status, respectively (Fig. 4A and B). The value of the percent thyroid uptake for each group measured by CLI is about one-fifth of that measured by γ-scintigraphy. The percent thyroid uptake of ^131^I measured from the two imaging modalities were well correlated and had a high linear regression (R^2^ = 0.9621) (Fig. 4C), indicating the feasibility and reliability of CLI for the measurement of ^131^I thyroid uptake.

## Discussion

In this study, we have demonstrated the feasibility of using CLI for quantification of ^131^I thyroid uptake function in mice. The results reveal a high linear correlation between ^131^I activity and the CL signals in the thyroid gland of the mice. In hypothyroid or hyperthyroid mice, CLI provides a reliable quantitative measurement of ^131^I thyroid uptake, which is comparable with the uptake measured by γ-scintigraphy and compatible with the thyroid function status of the mice.

The hypothyroid and hyperthyroid mice were induced by administration of MMI and rhTSH, respectively. Administration of rhTSH into normal subjects resulted in a significant increase in serum T4 at 8 h, reached to peak at 24 h and remained the high T4 level for at least 4 days^16^. Increased radioiodine uptake by normal subjects has also been observed at 6 and 24 hours after rhTSH treatment^17^. Serum T3 and T4 concentrations in euthyroid mice are strikingly increased at 6 hour after rhTSH treatment^18^. In the current study, the total T4 in euthyroid mice was 3.7 ± 0.9 μg/dl (n = 6), comparable with a range of 4 ~ 8 μg/dl for balb/c mice in earlier literatures^19, 20^ and with the data outlined by the Mouse Phenome Database at the Jackson laboratory. At 24 hours after two injections of rhTSH (totally 4 μg), serum T4 was effectively elevated by four folds (14.8 μg/dl). The effect of rhTSh on the radioiodine uptake of euthyroid mice has also been studied with ^125^I, which showed decreased uptake during the first 3 to 5 hours after treatment and then increased at 13 hours^18^. Based on these reports, 24 hours after rhTSH treatment was assumed to be the most suitable time point for the ^131^I imaging in mice. Notably, the mice with rhTSH stumulation showed a two-fold increase in ^131^I accumulation in thyroid glands as compared to that in control mice in this study. MMI has been used to treat hyperthyroidism caused by Graves’ disease for more than half a century, and is able to effectively induce hypothyroidism in euthyroid rodents^21, 22^. The mice were treated with ~1.6 mg (64 mg/kg) of MMI for 15 consecutive days. The serum T4 concentration (2.1 ± 0.4 μg/dl) was reduced to almost half of that in control mice (3.7 ± 0.9 μg/dl). ^131^I uptake by the thyroid gland measured by both CLI and γ-scintigraphy showed a decrease by one fifth of that of the euthyroid mice. In summary, hypothyroidism or hyperthyroidism of mice induced by MMI or rhTSH, respectively, is able to be monitored by ^131^I CLI with the same efficacy as γ-scintigraphy.

Obvious tissue attenuation of CL signal was seen in the animal experiments in this study. The percent thyroid uptake of ^131^I calculated from CL was about one fifth of that measured by γ-scintigraphy. The CL intensity is relatively weak and the most intense CL occurs in the spectrum of the UV and blue wavebands. UV and blue light are easily scattered and absorbed by biological tissues^23^. In addition, IVIS imaging system used in the current study is able to measure light signal with wavelength from 490 to 850 nm, which falls in the spectrum of green to red/near infrared light. Most CL signal cannot be detected by the optical device we used and consequently the percent thyroid uptake of ^131^I measured by CLI would be lower than by γ-scintigraphy. In the current study, 24 hours after administration of 18.5 MBq of ^131^I, the calculated accumulation of ^131^I in the hypofunctioning and hyperfunctioning thyroids from γ images was 0.5 and 5.9 MBq, respectively. This amount of ^131^I accumulation enables CLI to obtain enough photons to calculate the thyroid uptake which shows a high linear regression of correlation to the γ-scintigraphy, despite the remarkable attenuation of the CL signal. The dose of ^131^I calculated by the equation of minimum efficacious radiopharmaceutical dosage for scintigraphic imaging of small children and infants as (adult dosage)(child’s weight in Kg)/70 Kg, should be 0.15 μCi (0.006 MBq)^24^, which is about 0.03% of the administered dose of 500 μCi (18.5 MBq) in the current study. ^131^I has a physical half-life of 8.1 days and decays by beta emission. The major gamma emission is at 364 keV. The long half-life and the beta decay result in relatively high radiation on thyroid gland and other organs. The estimated absorbed radiation dose to the thyroid gland is 16,000 rad/mCi in the newborn, 800 rad/mCi in the adult^25^ and about 77,700 rad/mCi in rat^26^. The absorbed radiation dose to the mouse thyroid gland would be much higher. The dosage of ^131^I of 500 μCi (18.5 MBq) used in the current study might damage the thyroid gland and cause hypothyroidism of the animal in the long run. According to the results of the current study, we would recommend that 125 μCi (4.6 MBq) is the minimum dosage of ^131^I for optimal CLI of mouse thyroid gland.

In recent years, investigators have attempted to use CLI more effectively in clinical experiments by improving the image equipment. Spinelli *et al*. optimized the CCD detector for the near infrared (NIR) range, which contained a smaller number of Cerenkov photons, but had better tissue penetration ability. The sensitivity was improved up to 35%, and this advantage was more significant when the Cerenkov source is being imaged at a deeper location within the animal^27^. CCD systems with enhanced sensitivity for detecting low-light levels, such as intensified CCD (ICCD) and electron-multiplying CCD (EMCCD), have been used in recent studies^14, 28, 29^. These highly sensitive intensified CCD systems have recently been coupled to optical fiber allowing the demonstration of preclinical endoscopic surgery^30^. The results of a previous clinical study showed a good consistence with clinical PET examinations and the signals obtained by the endoscopic CLI to detect gastrointestinal malignant neoplasms^31^. These newly developed technology of optical imaging instruments are expected to improve both the quality of ^131^I imaging of thyroid gland and the measurement of ^131^I thyroid uptake by CLI as well.

In conclusion, we have demonstrated the feasibility of using CLI for quantitatively assessment of thyroid uptake function of radioiodide. CLI is able to quantify the ^131^I accumulation in the thyroid glands of mice with different functional status. The thyroid uptake of ^131^I measured by CLI is comparable to that obtained by γ-scintigraphy. In addition to nuclear medicine imaging modalities, CLI is useful to assess the thyroid uptake function of radioiodine in preclinical and basic research settings.
